# Susceptibility of Cooking Herbs to Stored-Product Moths

**DOI:** 10.3390/insects17020140

**Published:** 2026-01-26

**Authors:** Serena Malabusini, Alyssa Hidalgo, Perrine Noquet, Daria Patrizia Locatelli, Lidia Limonta

**Affiliations:** 1 Department of Food, Environmental and Nutritional Sciences (DeFENS), Università degli Studi di Milano, Via Celoria 2, 20133 Milan, Italy; alyssa.hidalgovidal@unimi.it (A.H.); daria.locatelli@unimi.it (D.P.L.); lidia.limonta@unimi.it (L.L.); 2 L’Institut Agro Dijon, 26, bd Docteur Petitjean, BP 87999, 21079 Dijon, Cedex, France; perrine.noquet@agrosupdijon.fr

**Keywords:** Lepidoptera, *Plodia interpunctella*, *Idaea inquinata*, infestation, secondary metabolites, survival, pest

## Abstract

Although herbs can contain several secondary metabolites potentially harmful to insects, dried herbs, like all stored foods, can be damaged by various insect pests. These pests have the potential to diminish both the quantity and the quality of food, and the consequences of infestation often only become noticeable once the pest has already become established. This study investigates the ability of two Lepidoptera pests, one polyphagous, *Plodia interpunctella*, and one selective, *Idaea inquinata*, to complete the postembryonic development on eleven dried herbs commonly used in cooking: dill (*Anethum graveolens* L.), basil (*Ocimum basilicum* L.), chervil (*Anthriscus cerefolium* (L.) Hoffm.), coriander (*Coriandrum sativum* L.), tarragon (*Artemisia dracunculus* L.), chives (*Allium schoenoprasum* L.), oregano (*Origanum vulgare* L.), parsley (*Petroselinum crispum* (Mill.) Fuss), sage (*Salvia officinalis* L.), savory (*Satureja hortensis* L.), and thyme (*Thymus vulgaris* L.). The results show that tarragon, sage, savory, oregano and thyme did not allow growth of either species, suggesting the presence of chemical compounds that limit larval growth or survival. In addition, the time required for both species to complete development was longer than that obtained on a standard diet, suggesting that the nutritional requirements and water content of the herbs are not as optimal as those of the standard diet. To conclude, *I. inquinata* could develop on basil, chervil, chives, coriander, dill, and parsley, while *P. interpunctella* could develop only on chervil, chives, coriander, and dill.

## Simple Summary

Like other stored foods, dried cooking herbs can be damaged by various insect pests, despite containing secondary metabolites that may be toxic to insects. These pests can affect the amount and quality of food, and the effects of infestation often do not become apparent until it has already taken hold. This study investigates the ability of two Lepidoptera pests—one polyphagous, *Plodia interpunctella*, and one selective, *Idaea inquinata*—to feed on eleven dried herbs frequently used in cooking. Tarragon, sage, savory, oregano, and thyme were found to inhibit the growth of both species, indicating the presence of chemical components that restrict larval growth or survival. Furthermore, both species can complete development on chervil, chives, coriander, and dill. However, it took longer than on a standard diet, suggesting a nutritional deficiency.

## 1. Introduction

Since ancient times, herbs have been used in cooking to enhance the flavor of food. More recently, it has been demonstrated that herbs exhibit antimicrobial properties against plant and human pathogens alike [1,2]. Indeed, essential oils derived from herbs have been shown to have insecticide properties [3,4,5,6,7,8,9]. Terpenes, phenolic acids, and flavonoids are some of the bioactive compounds responsible for insecticidal activity [10,11]. These secondary metabolites can act as repellents, antifeedant, or toxic substances to herbivorous insects [12,13]. However, some stored-product pests have developed the ability to survive on dried herbs, suggesting the evolution of physiological or behavioral adaptations that allow them to tolerate or overcome these chemical defenses.

Dried herbs, like all stored food, can be damaged by various pests, including insects. These pests can reduce the quantity and quality of food, and the consequences of infestation often only become apparent once the pests have already settled in. Polyphagous insects are the most problematic because they can develop on different substrates and can damage various food items stored in the same place. *Plodia interpunctella* (Hbn.) (*Lepidoptera Pyralidae*) is one such insect. This cosmopolitan and extremely polyphagous insect can feed on cereals, flour, nuts, dried fruit, dried vegetables [14], herbs, and dried meat. *P. interpunctella* can be used as a model species for studying insects’ adaptation to plant secondary compounds because of its high polyphagy and its ability to colonize different substrates. Investigating how this species develops on herbs with different nutritional and chemical compositions could reveal the mechanisms behind its remarkable ecological plasticity.

In contrast, *Idaea inquinata* (Scopoli) (*Lepidoptera Geometridae*) exhibits a narrower trophic niche and is a pest in haylofts and in barns [15,16], mainly associated with dry plant material such as herbs and medicinal plants [17,18]. This indicates a potential adaptation of this insect to the volatile-rich environment typical of these substrates; yet, its developmental performance on different herbs remains poorly understood.

This study investigates the ability of these two Lepidopteran pests to feed on eleven dried herbs that are commonly used in cooking by comparing their developmental performances. This will help us identify potential plant species that are more susceptible or resistant to infestation. Furthermore, we also explore how the nutritional value of the herbs may influence pest colonization in stored products.

## 2. Materials and Methods

### 2.1. Insect Rearing

The two species tested in this study were maintained in the laboratory rearing facility of the Department of Food, Environmental and Nutritional Sciences, Entomology Section, at the University of Milan (Italy). A specific standard diet was used for each species. The diet for *Plodia interpunctella* consisted of bran (16 g), wheat flour (15 g), corn flour (17 g), wheat germ (5 g), dried yeast (4 g), honey (19 g), and glycerin (24 g). The standard diet for *Idaea inquinata* contained the same ingredients in different quantities: 62 g of bran, 7 g of wheat flour, 8 g of corn flour, 4 g of wheat germ, 3 g of dried yeast, 7 g of honey, and 9 g of glycerin. Both species were kept in a thermostatic chamber at a temperature of 26 ± 1 °C and a relative humidity of 60 ± 5%. The nutritional characteristics of the diets are shown in Table 1 [19].

### 2.2. Herbs Used in the Experiment

The following eleven herbs were selected for the different trials: dill (*Anethum graveolens* L.), basil (*Ocimum basilicum* L.), chervil (*Anthriscus cerefolium* (L.) Hoffm.), coriander (*Coriandrum sativum* L.), tarragon (*Artemisia dracunculus* L.), chives (*Allium schoenoprasum* L.), oregano (*Origanum vulgare* L.), parsley (*Petroselinum crispum* (Mill.) Fuss), sage (*Salvia officinalis* L.), savory (*Satureja hortensis* L.), and thyme (*Thymus vulgaris* L.).

### 2.3. Rearing on Different Herbs

#### 2.3.1. Experimental Protocol

The eggs of *P. interpunctella* and *I. inquinata* were obtained, from the rearing systems, by placing newly emerged adults in a 1.7 L glass jar with a diameter of 10 cm and a height of 19 cm. The jar was closed with tulle and turned upside down in a Petri dish containing filter paper. Eggs aged 0–24 h were collected simultaneously for all the replicates and examined under a stereomicroscope to remove any damaged or irregular eggs. Groups of 50 eggs (randomly assigned to treatments) were placed on 10 g of one of the eleven herbs and on a standard diet in 15 cm Petri dishes. Five replicates were carried out for each herb and standard diet (Sd) for each Lepidoptera. The Petri dishes were kept under the same environmental conditions as the culture (26 ± 1 °C; 60 ± 5% R.H.).

Each replica was checked daily for 90 days. The number of new adults emerging each day was monitored. This enabled us to obtain data on the number of adults that emerge and developmental times. For each laid egg, developmental time was defined as the number of days from egg deposition to adult emergence.

#### 2.3.2. Statistical Analysis

Data were analyzed using generalized linear models with the statistical software R (version 4.5.0). Mean developmental time (days from egg deposition to adult emergence) and the number of emerged adults per replicate, when normally distributed, were analyzed using univariate analyses of variance (ANOVAs), and factors showing significant differences were subjected to post hoc Tukey’s tests with a type I error rate of 0.05. When data did not conform to the assumptions of ANOVA, non-parametric tests based on ranks were used. These analyses included only individuals that successfully completed development. The different herbs were fitted as a categorical factor in all analyses. In addition, the effect of different herbs on developmental time was assessed using parametric cohort survival analyses. Time to adult emergence was modeled using Weibull distributions with time-dependent hazard functions, with herb treatment included as a categorical predictor. Eggs that did not produce any adults were treated as censors [20,21,22].

### 2.4. Herb Analysis

Analyses of the herbs were conducted in duplicate, except for fiber, which was determined in triplicate, following AOAC procedures [23]: dry matter was gravimetrically determined by method 934.01, protein content (g/100 g DM) by the Kjeldahl approach (method 979.09 or 2001.11) using 6.25 as the nitrogen-to-protein conversion factor, and ash (method no. 967.05 or 923.03) by incineration at 600 °C using a Tactical 308 muffle (Gallenkamp, Cambridge, UK). Total lipid content was determined by the gravimetric method after Soxhlet extraction (Ba 3–38 method; [24]) using chloroform as a solvent. Fiber content was analyzed according to Aitkin et al. [25]. Total carbohydrates were computed by difference.

## 3. Results

### 3.1. Rearing on Different Herbs

The number of emerging adults per replica on each herb () was counted daily to assess the percentage of egg emergence after 90 days and life cycle dynamics starting from the egg to the adult stage. Neither species developed on tarragon, sage, savory, or thyme (no adults emerged in any of the replicates). Oregano prevented the development of moths (0 adults emerged from all 5 replicates of *I. inquinata*), except for one adult of *P. interpunctella* that emerged after 81 days in only one replica. In addition, this species did not develop on basil or parsley (no adult emergence for any of the *P. interpunctella* replicates), whereas *I. inquinata* did.

**Figure 1:**
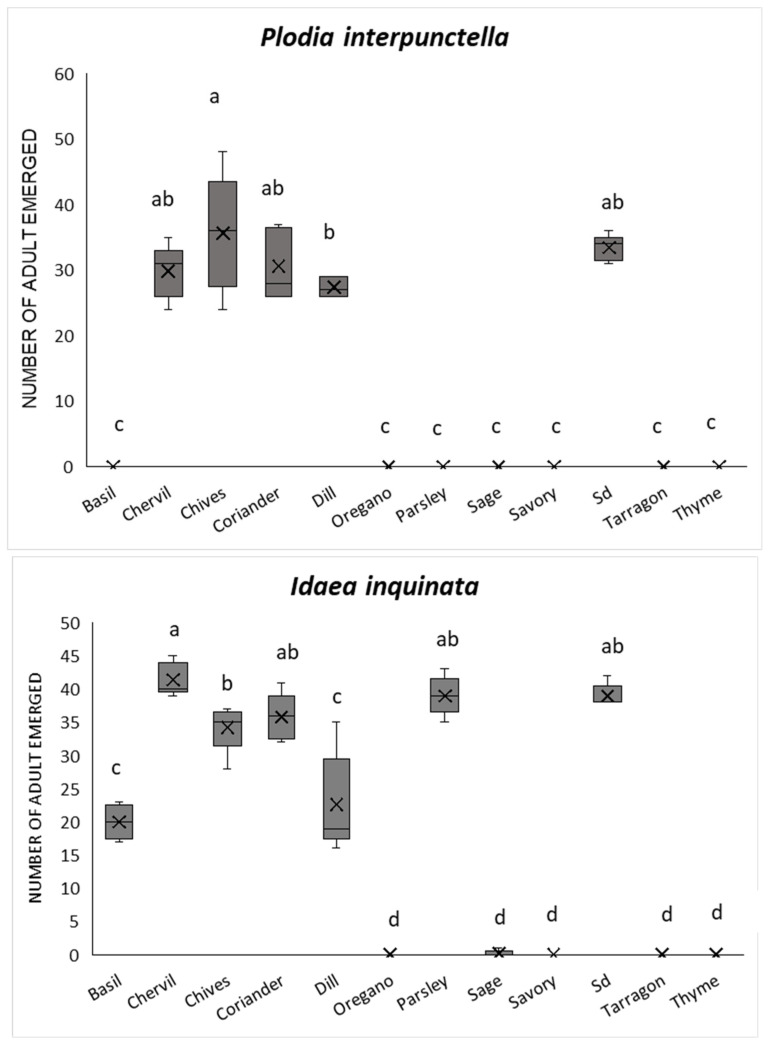
Box plots of number of adults of *Plodia interpunctella* and *Idaea inquinata* emerged on different herbs. Letters refer to the differences between diets (*p* < 0.05) using ANOVA and Tukey’s post hoc test; Sd, standard diet; “X” represents the mean.

**Figure 2:**
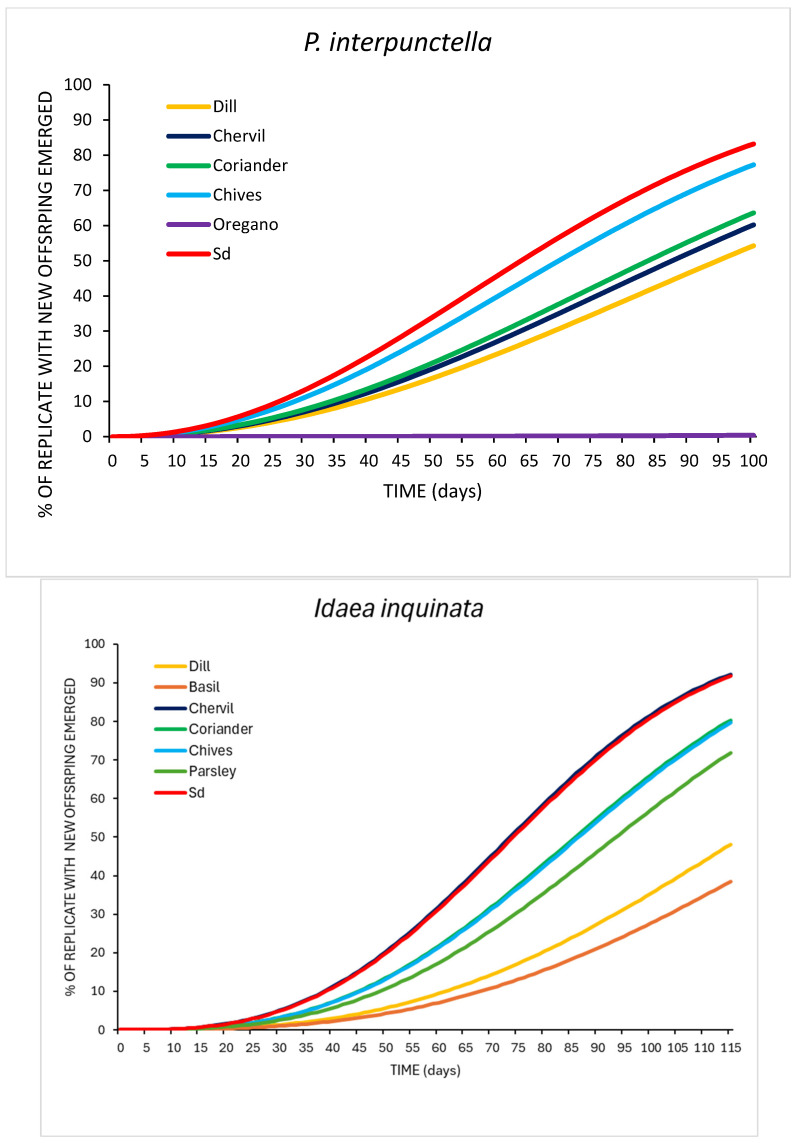
Cohort survival analysis of *Plodia interpunctella* and *Idaea inquinata* adult emergence, and Weibull model of time from egg to adult emergence, with replicates with no adults emerging treated as censors; Sd, standard diet.

A significantly different number of offspring was obtained on the different herbs and on the standard diets, for both species (*P. interpunctella*: F_11,48_ = 119.40; *p* < 0.001; *I. inquinata*: F_11,48_ = 186.18; *p* < 0.001). In detail, we found that a higher number of *P. interpunctella* adults emerged on chives, standard diet, coriander, and chervil: 35.6 ± 4.01, 33.4 ± 0.87, 30.6 ± 2.44, and 29.8 ± 1.83, respectively. The highest number of adults of *I. inquinata* was obtained on chervil, Sd, coriander, and parsley: 41.4 ± 1.12, 39.0 ± 0.77, 35.8 ± 1.59, and 39.0 ± 1.3, respectively (Figure 1). For both insect species, a higher percentage of new adult emergence was obtained on standard diet, chives, coriander, and chervil: between 71.2% and 59.6% for *P. interpunctella* and between 82.9% and 71.6% for *I. inquinata* (Figure 2). Cohort survival analyses showed that herb treatments significantly affected the time to adult emergence in both species (*P. interpunctella*: G_5_ = 531.45, *p* < 0.001, n = 1500; *I. inquinata*: G_6_ = 354.35, *p* < 0.001, n = 1750). Cumulative adult emergence curves for each treatment are shown in Figure 1.

**Figure 3:**
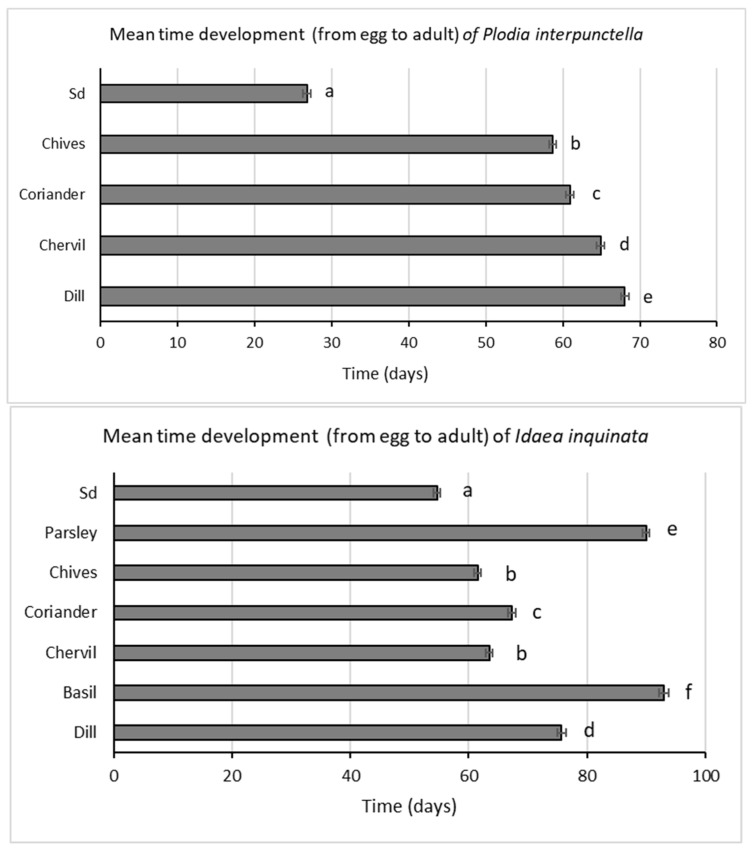
Mean time (±SE, indicated by error bars) of development (from egg oviposition to adult emergence) of *Plodia interpunctella* and *Idaea inquinata* on different herbs. Letters refer to the differences between diets (*p* < 0.05) using ANOVA and Tukey’s post hoc test; Sd, standard diet.

The mean developmental time differed significantly with diet in both moth species (*P. interpunctella*: F_4,787_ = 1201.21; *p* < 0.001; *I. inquinata*: F_6,1153_ = 511.43; *p* < 0.001). In detail, for both species, the fastest development took place on Sd: 26.8 ± 0.47 days for *P. interpunctella* and 54.6 ± 0.58 days for *I. inquinata*. By contrast, the slowest development was observed on basil (93.04 ± 0.81 days) for *I. inquinata*, excluding oregano, where only 1 adult emerged after 81 days, and on dill (68.1 ± 0.52 days) for *P. interpunctella* (Figure 3).

### 3.2. Herb Analysis

The major nutritional components of the eleven aromatic herbs tested showed interspecific variability in their proximate composition (Table 2). Water content ranged from 10.0 ± 0.02 g/100 g in chervil to 13.3 ± 0.09 g/100 g in savory, with most species exhibiting moisture levels around 11.1 g/100 g. The lipid content was relatively low in all herbs, varying between 4.0 ± 0.24 g/100 g in coriander and 11.1 ± 0.37 g/100 g in sage. Ash content, an indicator of total mineral matter, was highest in basil (16.2 ± 0.14 g/100 g) and lowest in chives (8.9 ± 0.04 g/100 g). Protein concentration varied considerably among species, ranging from 7.7 ± 0.09 g/100 g in sage to 27.4 ± 0.24 g/100 g in chervil. Fiber content showed pronounced variability, being lowest in dill and coriander (22.9 ± 0.87 g/100 g and 23.1 ± 1.00 g/100 g, respectively) and highest in sage (45.2 ± 0.21 g/100 g) and oregano (43.1 ± 0.32 g/100 g). Carbohydrate content (calculated by difference) ranged from 7.0 ± 1.28 g/100 g in basil to 26.5 ± 0.87 g/100 g in chives. Overall, the obtained values are consistent with the centesimal compositions reported by previous research [26].

## 4. Discussion

This study shows that the growth and survival of *Idaea inquinata* and *Plodia interpunctella* are affected by the dried herbs utilized as substrates. Neither species was able to complete development on tarragon, sage, savory, oregano, or thyme, suggesting the presence of nutritional or chemical components that limit larval growth or survival. The essential oils of these dried herbs contain monoterpenes such as carvacrol and thymol [27,28,29]. These monoterpenes exhibit significant biological activity in the form of repellence and deterrence, decreased palatability, growth inhibition through altered protein availability, enzyme inhibition, and direct toxicity [28,30], which may have prevented the moths from completing their development. The literature offers a variety of assays that demonstrate the toxicity of these plants’ essential oil extracts. In an olfactometer choice test, thymus showed 100% repellency against *P. intercunctella* [31] and adult mortality of *Rhyzopertha dominica* [32], while savory and oregano showed 100% larval mortality [28,33]. Conversely, Locatelli et al. [16] observed that 12% of *I. inquinata* adults emerged on oregano. It is worth considering that the secondary metabolites of herbs are influenced by cultivar, geographic origin, and environmental conditions [34,35,36]. With reference to sage, previous work has shown that extracts of this herb were extremely poisonous and had antifeedant properties against larvae of *Plutella xylostella* (L.) [9], *Agrotis ipsilon* (Hubn.) [37], and *Spodoptera littoralis* (Boisduval) [38]. On the other hand, our results show that *I. inquinata* and *P. interpunctella* were able to successfully complete their life cycle on dill, chives, coriander, and chervil, with survival rates comparable to those achieved on standard diet.

When standard diet was excluded, the highest emergence percentages were found to be 71% for *P. interpunctella* on chives and 82% for *I. inquinata* on chervil. The fastest development times were seen in chives for both species (*P. interpunctella*: 58.65 ± 0.45; *I. inquinata*: 61.5 ± 0.62). This suggests that these two herbs may provide ideal conditions for growth, especially for *I. inquinata*, which shows higher survival rates than *P. interpunctella*.

The ability of moths to survive on certain diets could be affected not only by monoterpene activity, but also by other elements in the diet, such as protein and carbohydrates [39,40,41] and by the amount of water [42]. In fact, the lower water content in the herb diet compared to the standard diet may have an impact on development, resulting in a longer developmental time [19,43,44,45].

The nutritional composition analysis supports these biological patterns. Herbs that allowed full development (e.g., chervil, coriander, chives, and dill) showed good levels of proteins, which are necessary for larval growth [46], as well as carbohydrates and fibers [47]. In contrast, herbs that prevented the development of these insects, such as sage and oregano, demonstrated remarkable levels of components such as high fiber or lipid content alongside low protein levels, which may limit digestibility and nutrient uptake.

The fastest development observed with the standard diet compared to all herbs indicates that even the most favorable plant substrates cannot fully replicate the optimal nutritional value of artificial diets. As stated by Dang et al. [48], nutritional deficiency results in fewer emerging adults and longer developmental times.

## 5. Conclusions

Pest infestations can occur on dried herbs, despite the presence of secondary metabolites that have insecticidal properties. This study demonstrated that *P. interpunctella* and *I. inquinata* can survive and develop from egg to adult on chervil, chives, coriander, and dill with a similar number of adults emerging compared to the standard diet. Conversely, both species failed to develop on oregano, savory, sage, thyme, and tarragon, likely due to the chemical characteristics of these herbs. The essential oils of these herbs contain monoterpenes that, as reported in previous research, could exhibit significant biological activity against insects: this may have prevented the two moths from developing. Nutritional characteristics also play a role, especially in developmental times, which was significantly slower in herbs compared to the standard diet. The nutritional profile of chervil, dill, coriander, and chives was found to be balanced, with protein levels ranging from intermediate to high (19–27%), and moderate levels of fiber and lipids. This suggests that these plants may be useful in supporting the growth of the moths’ larvae. Conversely, the highest fiber fractions and the lowest protein contents were shown by sage, oregano, and thyme, a pattern that may contribute to the reduced palatability or limited larval development that was observed in these substrates. Additionally, the presence of certain secondary metabolites could play a role in survival. Our findings suggest that some of these herbs can provide an adequate environment for the growth and development of *P. interpunctella* and *I. inquinata*. This emphasizes the importance of monitoring and controlling these pests throughout the supply chain of food products to ensure that food is safe and pest-free for consumers, and highlights how greater attention should be dedicated to the herbs that have shown the greatest susceptibility.

## Figures and Tables

**Table 1:** Nutritional characterization (% of each nutrient) of the artificial diets for *Idaea inquinata* and *Plodia interpunctella* (mean ± S.D.) [19].

Nutrients	*Idaea inquinata*	*Plodia interpunctella*
Proteins	13.9 ± 0.26	8.6 ± 0.05
Lipids	2.9 ± 0.12	2.0 ± 0.19
Starch	22.0 ± 0.79	29.3 ± 1.13
Soluble carbohydrates *	8.1 ± 0.27	13.8 ± 0.21
Total fiber	27.6 ± 1.01	9.7 ± 0.37
Ashes	3.6 ± 0.08	1.4 ± 0.02
Moisture content	12.8 ± 0.01	12.4 ± 0.09

* Sum of glucose, fructose, and sucrose.

**Table 2:** Chemical composition (g/100 g; mean ± standard error) of herbs.

	Water	Lipids	Ashes	Proteins	Fibers	CHO’s
Dill	11.1 ± 0.02	4.5 ± 0.42	13.4 ± 0.08	24.1 ± 0.31	22.9 ± 0.87	24.1 ± 1.29
Basil	10.9 ± 0.00	4.4 ± 0.19	16.2 ± 0.14	20.3 ± 0.25	42.1 ± 0.87	7.0 ± 1.28
Chervil	10.0 ± 0.02	4.8 ± 0.57	13.5 ± 0.01	27.4 ± 0.24	27.0 ± 0.03	17.4 ± 0.29
Coriander	10.5 ± 0.07	4.0 ± 0.24	15.7 ± 0.1	20.7 ± 0.17	23.1 ± 1.00	26.4 ± 1.75
Tarragon	10.7 ± 0.05	4.1 ± 0.38	10.9 ± 0.08	17.7 ± 0.00	35.0 ± 0.82	21.9 ± 1.69
Chives	12.0 ± 0.03	4.7 ± 0.24	8.9 ± 0.04	19.3 ± 0.14	29.1 ± 0.68	26.5 ± 0.87
Oregano	13.2 ± 0.02	6.6 ± 0.10	10.0 ± 0.15	9.6 ± 0.00	43.1 ± 0.32	17.7 ± 0.37
Parsley	10.8 ± 0.08	5.8 ± 0.17	12.5 ± 0.00	20.6 ± 0.11	25.7 ± 0.81	23.9 ± 0.40
Sage	11.6 ± 0.06	11.1 ± 0.37	9.6 ± 0.08	7.7 ± 0.09	45.2 ± 0.21	14.7 ± 0.65
Savory	13.3 ± 0.09	5.8 ± 0.36	14.6 ± 0.17	21.8 ± 0.09	36.7 ± 0.40	7.5 ± 0.45
Thyme	13.1 ± 0.00	4.9 ± 0.09	11.0 ± 0.03	16.5 ± 0.16	37.3 ± 0.58	17.1 ± 1.05

## Data Availability

The data presented in this study are available in Mendeley Data at https://doi.org/10.17632/5d3ddt9x6d.1, reference number [49].
